# Crosstalk Analysis of Delay-Insensitive Code in High-Speed Package Interconnects

**DOI:** 10.3390/mi14051033

**Published:** 2023-05-11

**Authors:** Bo Sun, Zhaoxin Xu

**Affiliations:** School of Integrated Circuits, Guangdong University of Technology, Guangzhou 510006, China

**Keywords:** delay-insensitive coding, signal integrity, interconnect

## Abstract

The development of integrated circuits has increased the size of chip interconnects, which has brought challenges to interconnect design in chip packages. The closer the spacing between interconnects, the higher the space utilization, which can cause severe crosstalk problems in high-speed circuits. In this paper, we applied delay-insensitive coding to the design of high-speed package interconnects. We also analyzed the effect of delay-insensitive coding on crosstalk improvement in package interconnects at 26 GHz for its high crosstalk immunity. Compared to the synchronous transmission circuit, the 1-of-2 and 1-of-4 encoded circuits designed in this paper can reduce crosstalk peaks by 22.9% and 17.5% on average at a wiring spacing of 1–7 μm, which can achieve closer wiring spacing.

## 1. Introduction

Advances in chip technology have led to the development of packaging technology. The interconnect part of electronic packages has received particular attention because of its essential impact on signal quality. An increase in interconnect size inevitably leads to a rise in wire delay, and the requirement for compactness of wiring leads to an increase in crosstalk, which is particularly evident in high-speed packages [1]. Three-dimensional packaging can effectively increase the number of wiring layers and shorten the interconnection length between layers [2], and the most direct way to solve crosstalk is to increase wiring spacing, which will increase the area cost [3]. Some crosstalk equivalent models can also provide us with more accurate analysis of crosstalk [4,5,6]. Scholars have researched how to improve the performance of interconnects in high-frequency packages by focusing on materials, processes, and circuits. At the material level, existing research focuses on the possibility of using new materials as interconnects with better electrical properties that can be partially used in specific applications [7,8]. The authors of [9] proposed a multilayer graphene nanoribbon interconnect with superior crosstalk immunity at advanced process nodes. At the process level, the studies presented in [10,11] used more advanced through-hole fabrication processes to obtain vias with smaller diameter and reduce the spacing between vias, and Jangam and Iyer (2021) [12] used a novel silicon interconnect structure (SI-IF) instead of conventional printed circuit boards, which has the advantages of fine pitch, scalability, and heterogeneous integration. At the circuit level, Shi et al. (2019) [13] proposed a compensation structure based on a third-order Butterworth low-pass filter to increase the bandwidth of the interconnect circuit; Braunisch et al. (2017) [14] introduced a method to improve crosstalk noise on short channels using drivers, although it requires additional wiring; and Madhuri and Sunithamani (2019) [15] proposed a faster and less error-prone analytical model for interconnect structures. The authors of [16,17,18,19] discussed the crosstalk characteristics of CMOS gate-driven interconnect structures and the effect of simultaneous switching on crosstalk. The authors of [20] discussed the performance of three-dimensional interconnects with carbon nanotubes. In [21,22,23], the frequency response and frequency-domain stability of interconnects were analyzed, and a new FDTD model for simulation analysis was proposed. The studies above have contributed to improving the performance of packaged interconnects at different levels. Still, they also illustrate that interconnect transmission wire design is currently tricky in high-frequency packages, and most interconnects need to be specially designed to reduce crosstalk and delay.

The delay increase due to the interconnection size brings serious timing problems. Traditional synchronous communication circuits in on-chip networks have become a thing of the past, and self-timed circuits using asynchronous communication have received widespread attention because they avoid dependence on the global clock [24]. The coding method in which the request signal is encoded in the data for transmission in self-timed circuits is delay-insensitive coding [25]. Delay-insensitive coding is widely used due to its advantages, such as clockless ness and crosstalk resistance, and the most current applications are 1-of-2 coding and 1-of-4 coding [26,27]. This approach requires encoding at the transmitter and decoding at the receiver, which inevitably brings delay and area cost [28].

For this reason, many researchers have worked on optimizing the coding and decoding circuits and enhancing their performance. The authors of [29] proposed an incomplete m-of-n coding that can reduce the delay caused by the coding and decoding circuits. The authors of [30] presented a method to reduce the hardware cost of a detection circuit. On the other hand, [31,32] conducted different studies to enhance circuit performance. In addition, some studies focused on analyzing the reliability of delay-insensitive circuits; the authors of [33] systematically investigated the impact of permanent faults on the QDI NOC (quasi-delay-insensitive networks-on-chip) and proposed new detection and recovery techniques. The application of delay-insensitive coding in high-speed packaged interconnects can effectively increase the interconnect performance and reduce the wiring spacing of interconnects due to its high crosstalk immunity, which is essential for simplifying the design of interconnects. However, no related studies have been performed. 

This study analyzed crosstalk in high-speed packaged interconnects with delay-insensitive coding and investigated its effect on crosstalk improvement. Compared to a synchronous transmission circuit, this coding method effectively reduces the crosstalk peak while reducing the wiring spacing of interconnects and increasing the density of interconnects, which can be achieved with a wiring spacing of 2 μm, considering only the BER.

## 2. Principle and Circuit Design

Delay-insensitive coding transmits the validity of the data implicitly along with the data. Then, it decodes the data at the receiving end, thus avoiding the requirement for delay between the request wire and the data wire. There are various delay-insensitive coding methods, such as dual-rail coding (1-of-2 coding), which uses two signal wires to transmit one bit of data, and the two wires transmit low and high logic levels, respectively. Each bit transmission involves only one of the two wires, and only one wire is allowed to transmit the signal at a time. The way this coding method works is shown in Table 1; the two wires are named dt and df, the low level of the data is 0, and the high level is 1. When both are low level 0, there is no data transmission at this time. When dt is 0, df is 1 when the transmission of logic is 0. When dt is 1, df is 0 when the transmission of logic is 1.

The electromagnetic field variations generated as the signal propagates along the transmission line generate noise on the surrounding lines, which usually occurs at the rising and falling edges of the signal, and the mutual capacitance and mutual inductance caused by the noise together form the crosstalk between the transmission lines [34]. The calculations of mutual capacitance (*C_c_*) and mutual inductance (*L_m_*) are shown in Equations (1) and (2) [25], where *w*, *h*, and *s* are the line width, line height, and line spacing, respectively; *t* and *ϵ* are the thickness and dielectric constant of the medium, respectively; *C_c_* is the self-capacitance; and *ϵ*_0_ and *μ*_0_ are the vacuum dielectric constant and magnetic permeability, respectively. It can be seen that when the spacing increases, both *C_c_* and *L_m_* decrease, and the crosstalk also decreases:

(1) $$C_{c}=\epsilon \left(0.03\frac{w}{h}+0.83\frac{t}{h}-0.07{\left(\frac{t}{h}\right)}^{0.222}\right){\left(\frac{s}{h}\right)}^{-1.34}$$

(2) $$L_{m}=\frac{\epsilon_{0}\mu_{0}}{2}\left(\frac{1}{C_{s}}+\frac{1}{C_{s}+2C_{c}}\right)$$

Compared to synchronous coding, a circuit using delay-insensitive coding allows only one transmission line to transmit signals at a time, thereby reducing the probability of simultaneous switching of adjacent wires. Therefore, the use of delay-insensitive coding allows better crosstalk control and noise reduction. In the analysis of crosstalk, the signal-to-noise ratio (*SNR*) is usually used to measure the relationship between the signal and noise, as shown in Equation (3), where *A* is the peak amplitude of the signal and $e_{v}$ is the noise amplitude [35]:

(3) $$SNR=20log\left(\frac{A}{\sqrt{2}e_{v}}\right)$$

**Figure 1:**
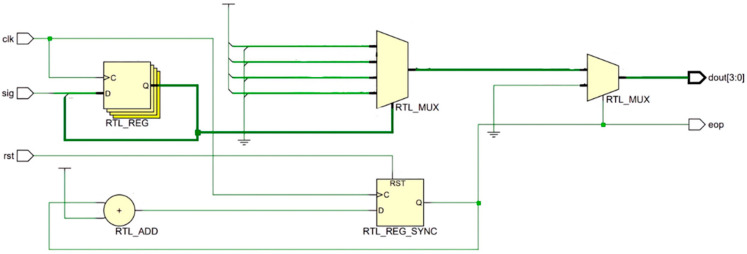
A 1-of-4 encoder.

**Figure 2:**
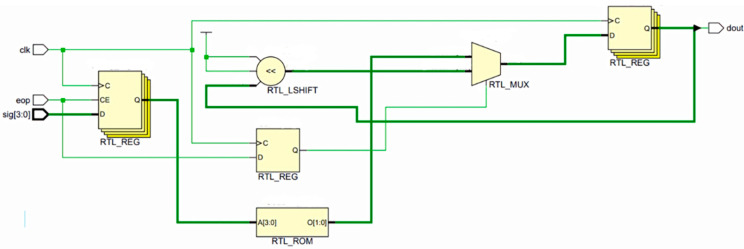
A 1-of-4 decoder.

The codec circuit is designed based on the principle of delay-insensitive coding, and the circuit is implemented in HDL (Hardware Description Language), with the core consisting of shift registers, selectors, and counters. Figure 1 and Figure 2 show the encoding and decoding circuits of quad-rail coding (1-of-4), which can realize the coding and decoding of two-digit binary data, respectively. 

**Figure 3:**
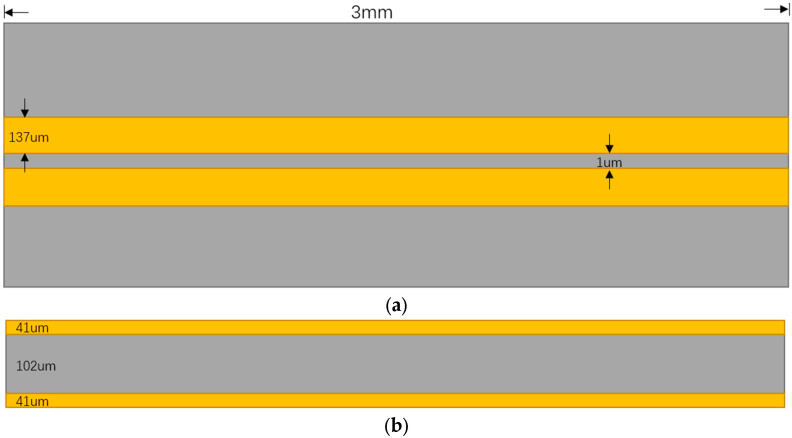
Transmission wire diagram: (**a**) top view and (**b**) cross-sectional view.

The circuit design was completed using Advanced Design System (ADS). The transmission wire was modeled as a microstrip wire built on a three-layer board with a signal alignment on the top layer, an FR4 layer in the middle, and a ground layer on the bottom layer. The transmission wire schematic is shown in Figure 3. The transmitter is the data transmitter, and the receiver is uniformly connected to a 50-ohm resistor. The signal frequency is 26 GHz, the amplitude is 1 v, the rise/fall time is 3.8 ps for a one-tenth cycle, the wire length is 3 mm, and the key design parameters are shown in Table 2. The standard of the logic level is shown in Table 3. V is the amplitude of the voltage.

**Figure 4:**
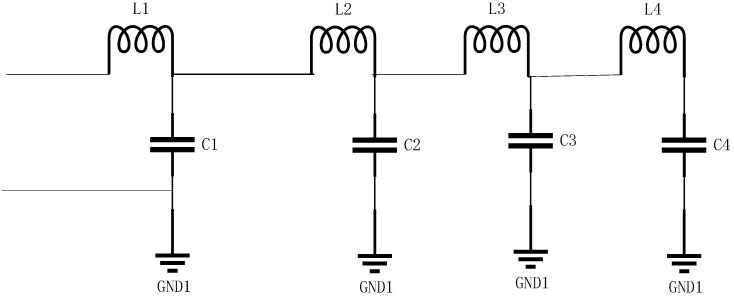
The physical structure of transmission lines.

The circuit diagram of the transmission line is shown in Figure 4.

As shown in Table 2, the line width of 137 μm is chosen with reference to the common widths used in package interconnect designs. The length of the interconnects and the thickness of the metal layers are also common lengths and thicknesses used in package interconnect designs. 

## 3. Circuit Crosstalk Analysis

Three sets of circuits were designed, including a conventional circuit using synchronous clock, a delay-insensitive 1-of-2 encoded circuit, and a 1-of-4 encoded circuit, all with the same design parameters.

### 3.1. The Synchronous Transmission Circuit

**Figure 5:**
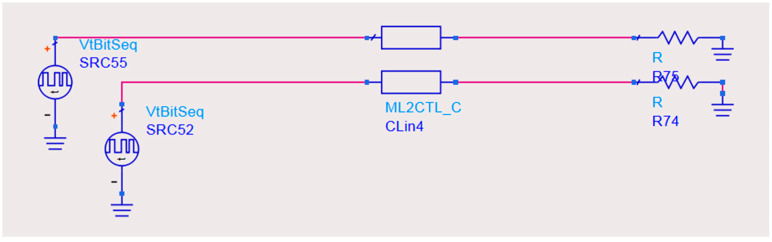
Synchronous transmission circuit.

**Figure 6:**
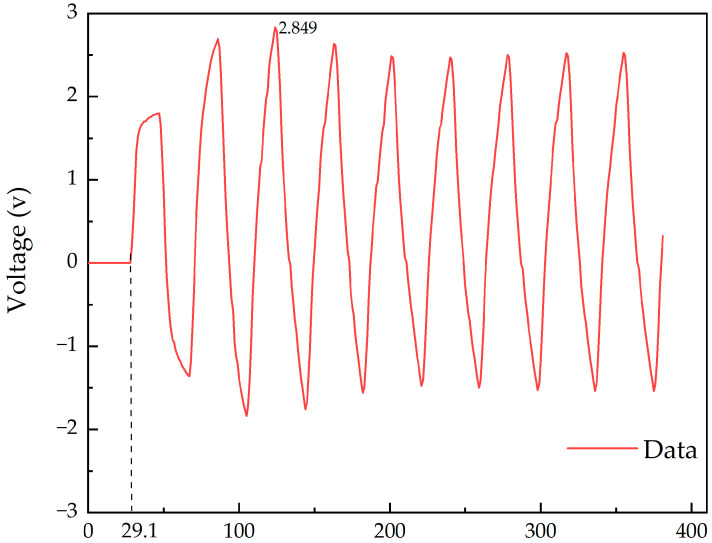
Signal waveform at the data receiver.

The circuit schematic is shown in Figure 5 and contains a clock wire and a data wire. The spacing between the transmission wires is set to 1 μm, which is ideally the closest spacing in a packaged interconnect, and the signal condition at the receiving end is shown in Figure 6. The amplitude of the crosstalk voltage can be obtained by subtracting the peak value of the voltage from the amplitude of the input voltage, and the transmission delay of the signal can be obtained based on the transmission time of half of a data cycle. As shown in Table 4, the transmission delay is 10.1 ps at 1 μm spacing, which is about 0.56 times the high level period. The delay is even greater when the wire is longer, causing a serious timing offset problem. In addition, with such a close spacing of 1 μm, the massive crosstalk can cause severe error codes, and the receiver does not correctly recognize the data of each cycle.

**Figure 7:**
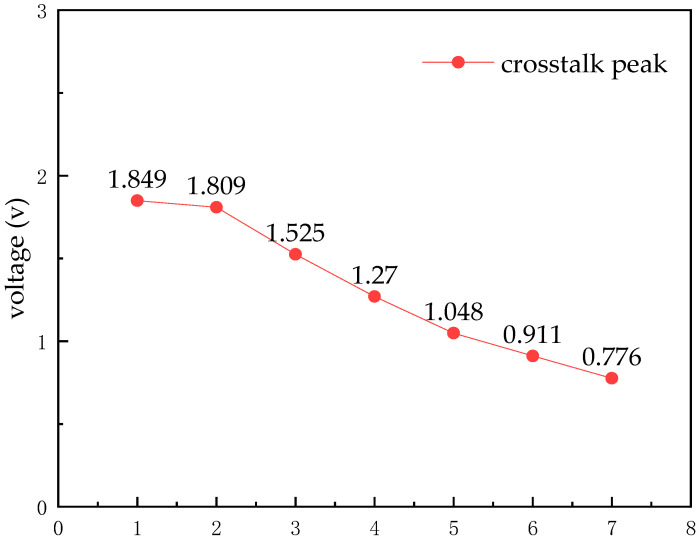
Crosstalk at different spacings.

The crosstalk is simulated for different wiring-spacing cases, and the crosstalk peak at the receiver side is observed. The results are shown in Figure 7. When the spacing s = 6 μm, there is a crosstalk voltage of 0.911 v, which causes the data at the transmitter side to not be received correctly. When s = 7 μm, the crosstalk voltage is 0.776 v, and no error code is generated. The minimum wiring spacing of the circuit is 7 μm.

### 3.2. The 1-of-2 Encoded Circuit

Conventional 1-of-2 coding transfers one bit of binary data over two data wires, which causes a decrease in throughput while avoiding latency effects. One trinary data bit is transmitted on two data wires to improve the circuit’s performance, which slightly increases the decoding cost but significantly improves the data transfer speed. The structure of the circuit is the same as the synchronous transmission circuit with two transmission wires, d0 and d1, and the data encoding method is shown in Table 5. Each wire is responsible for transmitting two bits of information. d0 is responsible for transmitting data 00 and 10. In effect, 00 is transmitted in the data wire by a high level of 1, and 10 is transmitted by low level of −1. At the receiving end, the identification of the high and low levels is mainly achieved by identifying the 1 and −1 in the first half-cycle, and the 0 in the second half-cycle acts as the end-of-packet (EOP) signal that represents the end-of-packet transmission. The EOP signal and the level signal in one cycle are both accurately recognized at the receiving end before the data of this cycle are correctly transmitted.

**Figure 8:**
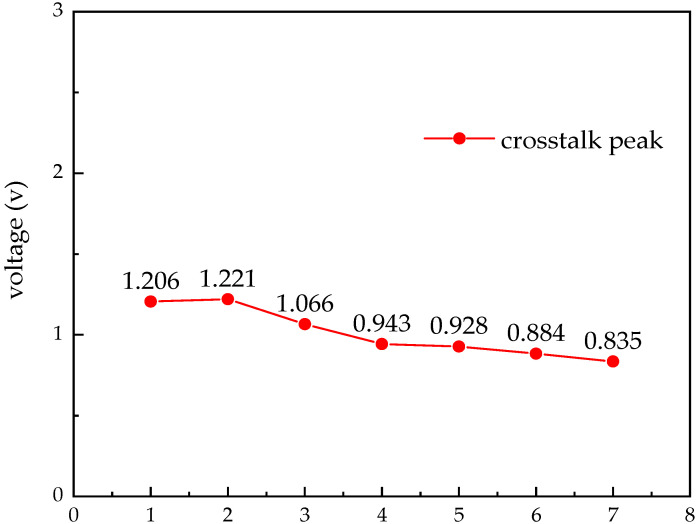
Crosstalk at different spacings.

Compared to the synchronous transmission circuit, the other settings are kept the same and only the coding method of the excitation source is changed. The simulation time is 0.4 ns, while gradually increasing the wiring spacing. The crosstalk peak is shown in Figure 8. It can be seen that when the wiring spacing is 6 μm, the crosstalk noise is 0.884 v, which is a large noise but does not produce error codes, indicating that the minimum wiring spacing of the circuit is 7 μm. Compared to the synchronous transmission circuit, with smaller wiring spacing, the crosstalk improvement effect is more obvious when s = 1 μm. At s = 1 μm, the crosstalk peak drops by 0.643 v, and the average crosstalk peak drops by 22.9% at a wiring spacing of 1–7 μm.

### 3.3. The 1-of-4 Encoded Circuit

**Figure 9:**
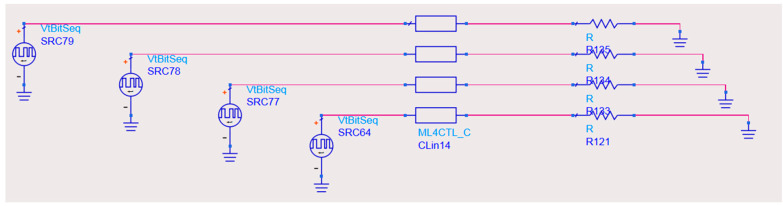
The 1-of-4 encoded circuit.

In the circuit shown in Figure 9, the four data wires are named d0, d1, d2, and d3. As with the 1-of-2 encoding, the 1-of-4 encoding changes the transmitted data from binary to trinary to significantly increase the circuit’s throughput rate. The four data wires are responsible for the two trinary data transfers. Each wire is responsible for two of 000, 001, 010, 011, 100, 101, 110, and 111, and the data codes are shown in Table 6.

**Figure 10:**
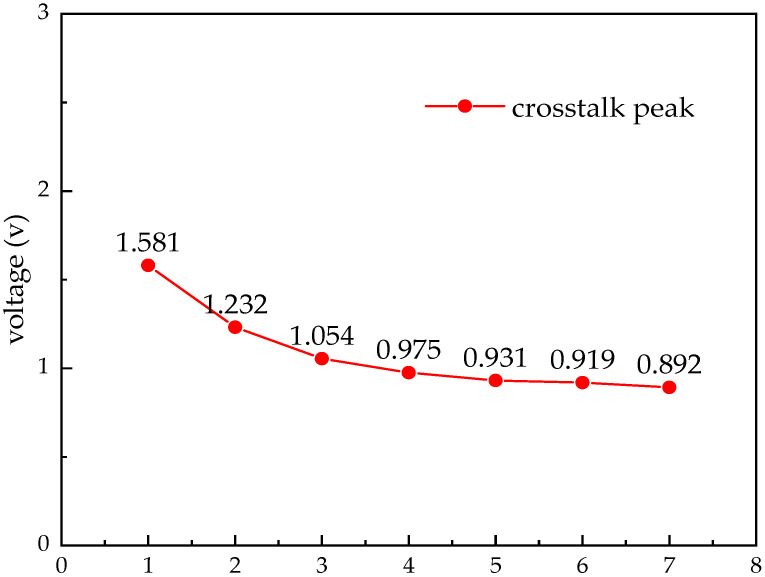
Crosstalk peaks at different wiring spacings.

The crosstalk peak is shown in Figure 10. Unlike the synchronous transmission circuit and the 1-of-2 encoded circuit, the crosstalk peak produces a large drop when the wiring spacing increases from 1 μm to 2 μm, after which the drop tends to level off with a minimum wiring spacing of 7 μm. The increase in wiring spacing leads to a complex situation between the transmitting and receiving wires, with the two data wires in between needing to withstand the noise generated by the two wires equally close together that are above and below. Compared to the synchronous transmission circuit, the average peak crosstalk drops by 17.5% at a wiring spacing of 1–7 μm.

### 3.4. Analysis and Comparison

**Figure 11:**
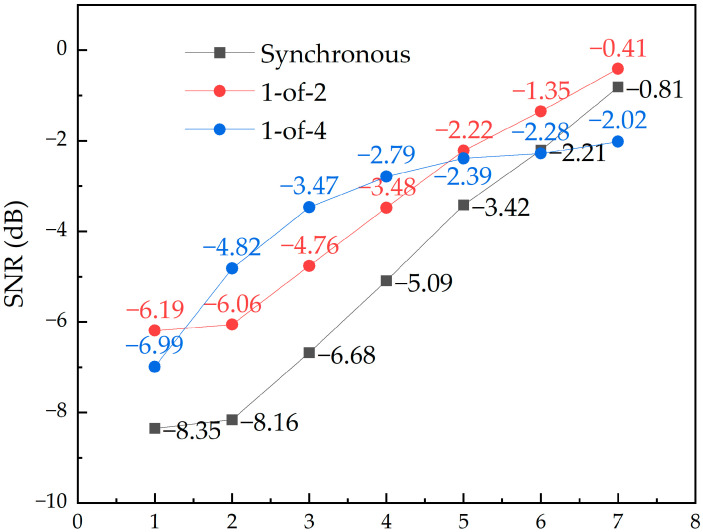
SNR at different spacings.

From Equation (1), the signal-to-noise ratio values for the three groups of circuits can be obtained, and $e_{v}$ is the amplitude of the noise, that is, the amplitude of the crosstalk, which can be obtained from the crosstalk peaks in Figure 7, Figure 8 and Figure 10. The results are shown in Figure 11. The 1-of-4 encoded circuit has the best signal quality among the three when the wiring spacing is 2–4 μm, and the remaining cases are more suitable for use with the 1-of-2 encoded circuit. The crosstalk situation is more complicated for the 1-of-4 encoded circuit, which results shown in Figure 11 do not form a certain pattern. If we look at the results of the synchronous transmission circuit and the 1-of-2 encoded circuit, we can see that the signal-to-noise ratio of the 1-of-2 encoded circuit is better than that of the synchronous transmission circuit at different wiring spacings.

#### 3.4.1. EOP Influence

For the 1-of-2 and 1-of-4 encoded circuits, the other settings are kept the same, and a separate EOP wire is set up where the EOP transmits a continuous string of 01 signals, where the “1” is the EOP signal. Since an additional transmission wire is added to transmit the EOP signal, it is necessary to consider whether the high and low levels of data information contained in each cycle are correctly transmitted through the data wire. The logic level standard remains unchanged, and the data are increased to 500 bits to analyze whether an EOP wire makes a difference for the two coding methods. The minimum wiring spacing standard is the case where no error codes are generated. As shown in Table 7, when an additional EOP wire is added, the minimum wiring spacing is improved for both 1-of-2 and 1-of-4 coding. This undoubtedly leads to increased wiring density, but the added EOP wire increases the area cost. If this area cost can be tolerated, an additional wire can act as an EOP wire in the circuit design.

#### 3.4.2. Crosstalk Analysis of Wiring with Different Wire Widths and Adjacent Layers

**Figure 12:**
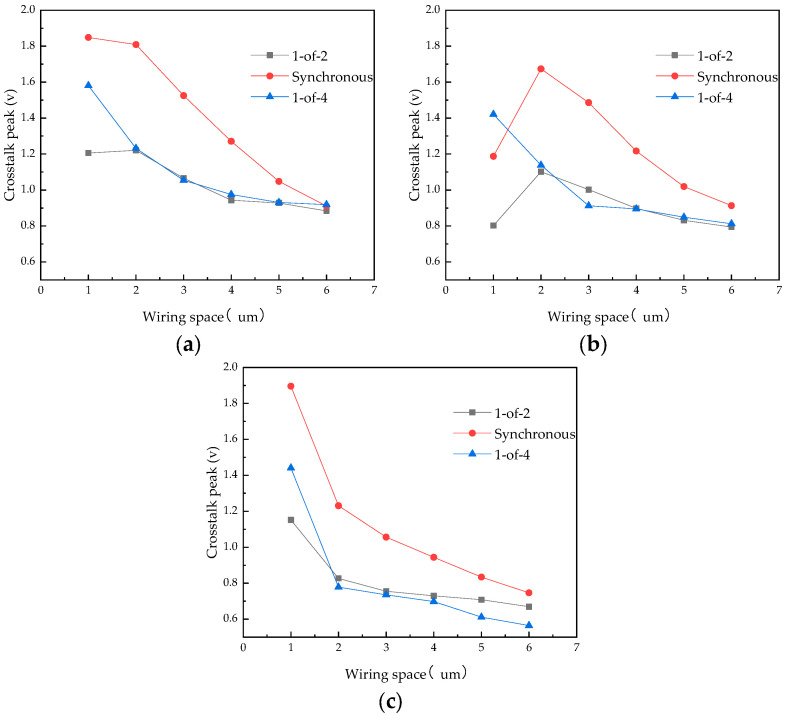
Crosstalk peaks with different wire widths: (**a**) w = 137 μm, (**b**) w = 80 μm, and (**c**) w = 40 μm.

The crosstalk of the three circuits was analyzed with different wire widths of 137 μm, 80 μm, and 40 μm for the 1-of-2 and synchronous trasmission circuits, and the results are shown in Figure 12. The wire widths of 137 μm, 80 μm, and 40 μm are all common widths used in package interconnect designs. With these three wire-width settings, the 1-of-2 coding and 1-of-4 coding improve the crosstalk better than the synchronous transmission circuit, and the closer the wiring spacing, the more obvious the improvement. When w = 80 μm, the crosstalk of the 1-of-2 encoded circuit and the synchronous transmission circuit increases and then decreases as the wiring spacing increases because when the spacing between the two wires is very small (1–2 μm in this case), the capacitive and inductive coupling offset part of the crosstalk, resulting in a crosstalk reduction.

A three-layer board is set up. The upper two layers are the signal layers, the bottom layer is the ground layer, the thickness of the dielectric layer is 102 μm, and the rest of the settings are consistent with the two-layer board. Two transmission wires are set up in the two signal layers, and the transmission wires are parallel at the top and bottom. The first layer signal wire has a signal input of 26 GHZ, and the second layer signal wire is not connected to any input. The maximum crosstalk voltages coupled to the lower layer alignments are 0.423 v and 0.339 v for the first layer signal wire using the 1-of-2 encoding and synchronous input signals, respectively. This indicates better crosstalk suppression can be obtained using delay-insensitive coding when wiring in different layers.

The structure proposed in this paper can be practically fabricated, and although a wiring pitch of a few μm is difficult to achieve in FR4, it can be fabricated in silicon substrates, and performing physical verification is the next focus of our research.

## 4. Conclusions

This paper presents a method to apply delay-insensitive coding to improve the crosstalk effect of high-speed packet interconnects and to enhance the circuit performance by increasing the data throughput rate. Compared to the synchronous transmission circuit, the 1-of-2 and 1-of-4 encoded circuits designed in this paper can reduce crosstalk peaks by 22.9% and 17.5% on average at a wiring spacing of 1–7 μm, which can achieve closer wiring spacing. If one is willing to pay a particular area cost, the wiring density can also be increased significantly by adding EOP wires, where 3 μm and 2 μm wiring spacing can be achieved for the 1-of-2 and 1-of-4 coding, respectively. This study can be applied to interconnect designs in high-speed packages.

## Figures and Tables

**Table 1:** The 1-of-2 code.

	dt	df
**Empty (“E”)**	0	0
**Valid (“0”)**	0	1
**Valid (“1”)**	1	0

**Table 2:** Key design parameters.

**Relative Permittivity**	**4.4**	**Frequency**	**26 GHz**
**Conductivity**	**5.80 × 107**	**Rise/Fall Time**	**3.8 ps**
**Thickness of metal layer**	41 μm	**Length of transmission wires**	3000 μm
**Thickness of substrate**	102 μm	**Width of transmission wires**	137 μm

**Table 3:** Standard of logic level.

**Logic 1**	**V > 0.9 v**
**Logic 0**	−0.1 v < V < 0.1 v

**Table 4:** Signal quality at the receiver.

**Transfer Delay**	**10.1 ps**
**Crosstalk peak**	1.849 v
**Bit error rate**	100%

**Table 5:** The 1-of-2 code.

Information Transferred	d0	d0	d1	d1
**00**	1	0	0	0
**01**	0	0	1	0
**10**	0	−1	0	0
**11**	0	0	0	−1

**Table 6:** The 1-of-4 code.

Information Transferred	d0	d0	d1	d1	d2	d2	d3	d3
000	1	0	0	0	0	0	0	0
001	0	0	1	0	0	0	0	0
010	0	0	0	0	1	0	0	0
011	0	0	0	0	0	0	1	0
100	0	−1	0	0	0	0	0	0
101	0	0	0	−1	0	0	0	0
110	0	0	0	0	0	−1	0	0
111	0	0	0	0	0	0	0	−1

**Table 7:** Impact of adding EOP wire for 1-of-2 and 1-of-4 coding.

		Minimum Wiring Spacing (μm)
**1-of-4 code**	With EOP wire	2
	No EOP wire	7
**1-of-2 code**	With EOP wire	3
	No EOP wire	6

## Data Availability

Data are available from the authors upon reasonable request.
